# Leveraging Artificial Intelligence in Marketing for Social Good—An Ethical Perspective

**DOI:** 10.1007/s10551-021-04843-y

**Published:** 2021-05-26

**Authors:** Erik Hermann

**Affiliations:** Wireless Systems, IHP - Leibniz-Institut für innovative Mikroelektronik , Frankfurt (Oder), Germany

**Keywords:** Artificial intelligence, Marketing, Ethics, Social good, Well-being

## Abstract

Artificial intelligence (AI) is (re)shaping strategy, activities, interactions, and relationships in business and specifically in marketing. The drawback of the substantial opportunities AI systems and applications (will) provide in marketing are ethical controversies. Building on the literature on AI ethics, the authors systematically scrutinize the ethical challenges of deploying AI in marketing from a multi-stakeholder perspective. By revealing interdependencies and tensions between ethical principles, the authors shed light on the applicability of a purely principled, deontological approach to AI ethics in marketing. To reconcile some of these tensions and account for the AI-for-social-good perspective, the authors make suggestions of how AI in marketing can be leveraged to promote societal and environmental well-being.

## Introduction

Artificial intelligence (AI) is not just a technology but a powerful force reshaping and benefiting societies by reducing costs and risks, increasing consistency and reliability, and providing new solutions to complex problems (Taddeo & Floridi, 2018). AI systems and applications have become pervasive across industries and sectors (Campbell et al., 2020) and also bring forth diverse opportunities for marketing strategy and actions (e.g., Huang & Rust, 2021b) as well as customer experience (e.g., Puntoni et al., 2021), relationships (e.g., Libai et al., 2020) and engagement (Kumar et al., 2019). The increasing computational power, data availability and intensity, context awareness, and emotional-sensing capabilities of AI allow to tailor customized and personalized offerings, and establish and maintain responsive customer interactions and relationships with experiential value (e.g., Grewal, Hulland, et al., 2020; Huang & Rust, 2021b; Ma & Sun, 2020; Puntoni et al., 2021). However, the substantial and growing scale and scope of consumer data feeding AI systems, the level of AI (emotional) intelligence, and AI-driven sales and consumption increases raise ethical controversies and challenges (e.g., Davenport et al., 2020; De Bruyn et al., 2020; Vlačić et al., 2021). Among other things, AI applications and systems can be discriminatory in various respects. On the customer level, discrimination can arise from customer prioritization based on demographic and economic factors (e.g., Libai et al., 2020) and targeting (e.g., Matz & Netzer, 2017) or alienation (e.g., Puntoni et al., 2021) of vulnerable consumer groups. On the company level, market share concentration through AI-enabled e-commerce platforms (e.g., Lee & Hosaganar, 2019) and unequal representation on them (e.g., Milano et al., 2021) can disadvantage some companies while privileging others. Such discriminatory treatments can reinforce and exacerbate existing economic and societal inequalities. Thus, ethical challenges related to AI in marketing can emerge on the customer, company, and societal levels. However, the discussion of ethical issues of AI in marketing is partly anecdotal and has hitherto focused on specific AI applications or aspects thereof and/or single ethical principles, for example, explainability (e.g., De Bruyn et al., 2020; Huang & Rust, 2021b; Rai, 2020) or privacy (e.g., Davenport et al., 2020; Kumar et al., 2019; Puntoni et al., 2021).

Given the substantial advancement and increasing prevalence of AI as well as its encompassing impact on the individual, economic, and societal levels, the debate on ethical principles and values guiding AI development and use has gained center stage (e.g., Cowls et al., 2021; Farisco et al., 2020; Floridi et al., 2018, 2020; Hagendorff, 2020; Jobin et al., 2019; McLennan et al., 2020; Mittelstadt, 2019; Mittelstadt et al., 2016; Morley et al., 2020; Stahl et al., 2021). To date, the AI ethics landscape is rather fragmented and entails recurring principles (Jobin et al., 2019) that are of high-order, deontological nature (Hagendorff, 2020). Translating these principles into business practice by simultaneously accounting for different stakeholder interests might demand tradeoffs, for instance, between need satisfaction due to personalization of offerings and privacy (e.g., Rust, 2020) or between customer prioritization and non-discrimination (e.g., Libai et al., 2020). That becomes particularly important when the objectives of AI should be to promote social good (*beneficence*) and prevent any harm (*non-maleficence*)—the call for and stance of AI for (social) good (Cowls et al., 2021; Floridi et al., 2018, 2020; Taddeo & Floridi, 2018).

To the best of our knowledge, our conceptual analysis is the first study to systematically apply the ethical principles related to AI to AI applications in marketing from a multi-stakeholder perspective. Our systematic conceptual assessment of the ethics of AI in marketing is informed by a comprehensive review of the literatures on both AI ethics and AI in marketing and provides two important contributions to both streams of research. First, we reveal interdependencies and tensions of ethical principles in dependence of the stakeholders concerned. Particularly, the principles *beneficence* and *non-maleficence* are interrelated and judged differently across the customer, company, and societal and environmental levels. We further identify *explicabilit*y (i.e., intelligibility and accountability) as enabling ethical principle. Moreover, ethical challenges and interdependencies are likely to intensify with increasing levels of intelligence and humanization of AI. To still harness and not miss opportunities provided by AI in marketing, the prevailing principled, deontological approach to AI ethics should be supplemented by a utilitarian perspective weighing benefits and costs across stakeholders. Second, we provide insights of how AI in marketing can be leveraged to promote social and environmental well-being and thus to reconcile the ethical principles of *beneficence* and *non-maleficence*. Our suggestions add knowledge to the scholarly work on AI for social good and sustainable consumption and marketing. The AI-for-social-good perspective stresses that AI-based solutions have the potential to tackle societal problems (e.g., Floridi et al., 2020)—among them, sustainable development as a focal challenge and objective of our time (Vinuesa et al., 2020). Given that marketing and consumption are part of our everyday lives, AI in marketing following the AI-for-social-good perspective can strive for and substantially contribute to sustainable development. In light of the environmental imperative (Kotler, 2011) and in the tradition of social marketing which its 50-year history (Kotler & Zaltman, 1971), companies increasingly pursue the transition to sustainable business and marketing practices (White et al., 2019). In the same vein, customers are concerned with the environmental and social impact of their purchases (Vadakkepatt et al., 2021) and demand sustainable products and services (Kotler, 2011). Therefore, AI in marketing that fosters environmental and social good can create win–win–win situations for companies, customers, and society at large (Vlačić et al., 2021).

The remainder of our study is structured as follows. After delineating our methodological approach and briefly illustrating the role and uses of AI in marketing, we present an overview of the rapidly expanding research on AI ethics. Afterward, we consolidate both perspectives by applying selected ethical principles to AI applications in marketing. We conclude our investigation with suggestions of how to harness AI in marketing for promoting societal and environmental well-being and with directions for future research.

## Methodology

To identify relevant scholarly work on AI ethics and AI in marketing, we conducted a systematic search of published papers. First, we performed a keyword search of electronic databases (Web of Science, EBSCO, and Google Scholar) using the following keywords: “ethic*,” “guidelines,” “principles,” “framework,” (for AI ethics) and “marketing,” “service,” “retailing,” “consumer,” “customer,” (for AI in marketing) each in combination with “artificial intelligence,” “AI,” “artificial,” “machine learning,” “algorithm*,” “robot*.” Second, we searched review and seminal articles in both fields (e.g., Davenport et al., 2020; Huang & Rust, 2021b; Kaplan & Haenlein, 2019; and Floridi et al., 2018; Jobin et al., 2019; Mittelstadt, 2019, respectively), examining their references and applying an ancestry tree search by screening all papers citing these articles. Third, we performed manual search of journal outlets that turned out to be major sources for journal articles dealing with AI in marketing and business (e.g., *Journal of the Academy of Marketing Science*, *Journal of Business Research*, *Journal of Interactive Marketing*, *Journal of Service Research*, *Journal of Marketing*; and particularly special issues on AI or robots in these journals) and AI ethics (i.e., *Ethics and Information Technology*, *Minds and Machin*es, *Nature Machine Intelligence*, *Science and Engineering Ethics*). This search procedure and screening of abstracts and titles lead to the selection of 300 potentially eligible articles (148 for AI in marketing and 152 for AI ethics), which were then reviewed in more detail. The systematic examination of the literatures on AI ethics and AI in marketing allows us to identity the focal areas of application of AI in marketing and to map the most relevant and appropriate ethical principles related to AI. Both are then synthesized to inform our conceptual analysis of the ethical concerns of AI in marketing from a multi-stakeholder perspective.

## AI in Marketing

AI can be conceptualized as “the use of computational machinery to emulate capabilities inherent in humans” (Huang & Rust, 2021b, p. 31) and refers to “programs, algorithms, systems or machines that demonstrate intelligence” in its simplest sense (Shankar, 2018, p. vi). In more methodological terms, AI can be defined as “a system’s ability to correctly interpret external data, to learn from such data, and to use those learnings to achieve specific goals and tasks through flexible adaptation” (Kaplan & Haenlein, 2019, p. 17). However, one should not assume that definitions of AI will be permanently stable given the conceptually challenging and changing nature of AI technologies (Stahl et al., 2021). AI in marketing has become increasingly important and is breaking new grounds in marketing research, strategy, and actions, customer relationships and experience (Davenport et al., 2020; Hoyer et al., 2020; Huang & Rust, 2021b; Kumar et al., 2019; Libai et al., 2020; Liu et al., 2019; Mustak et al., 2021; Puntoni et al., 2021). As the prevalence and diversity of AI advancements and applications is constantly growing across industries and sectors (Campbell et al., 2020; Haenlein & Kaplan, 2021; Kaplan & Haenlein, 2019), so do AI applications in marketing. To name but a few, AI is employed in and (re-)shaping services (e.g., Castillo et al., 2020; Huang & Rust, 2018, 2021a; Klaus & Zaichkowsky, 2020; Lin et al., 2021; McLeay et al., 2021; Mende et al., 2019; Wirtz et al., 2018; Xiao & Kumar, 2021), retailing (e.g., de Bellis, & Johar, 2021; Guha et al., 2021; Shankar, 2018), customer experience (e.g., Ameen et al., 2021; Hoyer et al., 2020; Puntoni et al., 2021), and customer relationships, engagement, and decision-making (e.g., Dellaert et al., 2020; Huang & Rust, 2021a; Kumar et al., 2019; Libai et al., 2020; Youn & Jin, 2021). At the core, AI applications aim at fine-grained and data-driven personalization and customization of products, services, and the marketing mix variables (e.g., Davenport et al., 2020; Huang & Rust, 2021b; Tong et al., 2020) along the entire customer journey (e.g., Hoyer et al., 2020) and service process (e.g., Huang & Rust, 2021a) to engage customers and optimize experiential value (e.g., Hoyer et al., 2020; Kumar et al., 2019; Puntoni et al., 2021).

To specify and systematize the opportunities of deploying AI in marketing, Huang and Rust (2021b) developed a three-stage strategic planning framework based on the marketing research–marketing strategy–marketing action cycle and three levels of AI intelligences, that is, mechanical, thinking, and feeling AI. While mechanical AI entails automation of repetitive and routine tasks, thinking AI relates to processing data for new insights and decision-making, and feeling AI refers to interactions with humans or analyzing human feelings and emotions. Huang and Rust (2021b) identified the following opportunities to leverage and benefit from AI in marketing:

1. Mechanical AI for data collection (marketing research), segmentation (marketing strategy), and standardization (marketing action).
2. Thinking AI for market analysis (marketing research), targeting (marketing strategy), and personalization (marketing action).
3. Feeling AI for customer understanding (marketing research), positioning (marketing strategy), and relationization (marketing action).

Davenport et al. (2020) also proposed a framework to foster the understanding and anticipation of the AI’s future impact on marketing and business. They proposed the following three AI-related dimensions: the level of intelligence (i.e., task automation versus context awareness), the task type (i.e., analysis of numbers versus non-numeric data such as text, voice, images, or facial expression), and whether the AI is embedded in a robot (i.e., virtuality–reality continuum). In a similar vein, Kaplan and Haenlein (2019) classified AI applications according to the level of intelligence into analytical AI (cognitive intelligence), human-inspired AI (cognitive and emotional intelligence), and humanized AI (cognitive, emotional, and social intelligence). The level of intelligence of AI as focal and recurring classification criterion relates to immense opportunities to harness AI by maximizing customer orientation and interaction, but it simultaneously poses challenges.

That is, AI systems that are humanized or emotionally intelligent do not come without ethical controversies (e.g., Belk, 2020; De Bruyn et al., 2020). Ethical questions are also raised in terms of explainability (e.g., De Bruyn et al., 2020; Rai, 2020), privacy (e.g., Davenport et al., 2020; Kumar et al., 2019), and trustworthiness (e.g., Glikson & Wolley, 2020), among others. Before we delve into the ethical questions related to AI in marketing, we shed light on the overarching debate on AI ethics.

## AI Ethics

The discourse on moral and ethical implications of AI dates back from 1960 (Samuel, 1960; Wiener, 1960). The tremendously intensifying development, use, and (societal) impact of AI in recent years has sparked calls for and discussions of accompanying ethical guidelines: “the ethical debate has gone mainstream” (Morley et al., 2020, p. 2141).

In a comprehensive review, Jobin and et al. (2019) content-analyzed the principles and guidelines for ethical AI issued by private, public, and research institutions. Remarkably, no single ethical principle is referenced in all 84 documents being analyzed. However, there is convergence around the principles *transparency*, *justice and fairness*, *non-maleficence*, *responsibility,* and *privacy*, which are featured in more than half of all documents. Among these principles, *transparency* constitutes the most prevalent one with references in 73 out of 84 documents, followed by *justice and fairness* (referenced in 68 documents), *non-maleficence* (referenced in 60 documents), *responsibility* (referenced in 60 documents)*,* and *privacy* (referenced in 47 documents).

Besides, Jobin et al. (2019) draw the following noteworthy conclusions. First, the prevalence of *transparency* could be partly explained by the reasoning that “is not an ethical principle in itself but a proethical condition for enabling or impairing other ethical practices or principles”, as suggested earlier by Turilli and Floridi (2009, p. 105). Second, the frequent occurrences of *justice* and *fairness*, *non-maleficence*, and *privacy* reflect a cautious view on potential risks of AI. Third, the more frequent references to *non-maleficence* as compared to *beneficence* imply the moral obligation to avoid any negative impact of AI and could imply a certain negativity bias. Fourth, the principle *trust* constitutes a critical ethical issue in AI governance. However, it is not without opposition and ambiguity, particularly, whether *trust* is a principle in itself or rather an outcome of other foundational principles (e.g., Floridi, 2019; Glikson & Wolley, 2020; Ryan, 2020; Thiebes et al., 2020). Although the principle *solidarity* refers to redistributing the benefits of AI to not jeopardize social cohesion (Jobin et al., 2019), it is featured in only 6 out of 84 documents. In light of significant differences in distributions of wealth and incomes within and between countries and economies (e.g., Piketty, 2014, 2020), prosperity and burdens created by AI should be shared to avoid further inequalities. That is, *solidarity* should be considered as a focal ethical principle of AI (Luengo-Oroz, 2019).

The principles *solidarity* and *beneficence* already hint at the need to harness the advantageous effects of AI on a societal level. Hence, the discourse on AI ethics also focuses ethical frameworks for AI for (social) good (Floridi et al., 2018, 2020; Taddeo & Floridi, 2018). In this context, Floridi et al. (2018) condensed five ethical principles: *beneficence*, *non-maleficence*, *autonomy*, *justice*, and *explicability*.

While *beneficence* entails to the promotion of well-being as well as social, environmental, and common good (Jobin et al., 2019; Thiebes et al., 2020), the tenet of *non-maleficence* cautions against the potentially negative aspects of AI. It emphasizes the importance of safety, security, and privacy as well as the prevention of risks and any harm—both accidentally/unintentionally (overuse) and deliberately (misuse) caused (Floridi et al., 2018; Jobin et al., 2019). Although *beneficence* and *non-maleficence* seem logically equivalent, they are not opposite ends of a continuum but coexist (Floridi et al., 2018). The principle *autonomy* refers to self-determination and the power to and whether to decide in an uncoerced way. That is, it concerns balancing human and AI agency and decision-making power (Floridi et al., 2018; Morley et al., 2020). *Justice* advocates fairness and the avoidance of unwanted/unfair biases and discrimination, also amending past inequalities (Jobin et al., 2019; Morley et al., 2020; Thiebes et al., 2020). *Justice* further relates to sharing benefits and prosperity and fostering solidarity (Floridi et al., 2018). Thus, the conceptual scope encompasses the *solidarity* principle, as opposed to its infrequent occurrence in official documents as indicated above (Jobin et al., 2019). Finally, *explicability* (also often conceptualized as *transparency*) means intelligibility, that is, how AI works (the epistemological sense), and accountability, that is, who is responsible for the way AI works (the ethical sense). It complements and enables the preceding four principles. In other words, understanding of the functionalities (i.e., intelligibility) and responsibilities (i.e., accountability) informs evaluations of and judgments of the other principles by facilitating understanding if and how AI benefits or harms individuals and society (*beneficence* and *non-maleficence*), anticipating AI systems’ predictions to decide about human and AI agency (*autonomy*) and ensuring accountability in case of failures or biases (*justice*) (Floridi et al., 2018; Thiebes et al., 2020). This is in line with the reasoning that *transparency* constitutes a pro-ethical condition for enabling other ethical principles (Turilli & Floridi, 2009). *Explicability* (particularly, intelligibility) gains in importance against the backdrop of the black box nature and opacity of AI systems and applications (e.g., Ananny & Crawford, 2018; Milano et al., 2020; Mittelstadt et al., 2016; Rudin, 2019; Thiebes et al., 2020), since black box AI could thwart evaluations of *beneficence*, *non-maleficence*, *justice*, and *autonomy*.

The first four principles are akin to bioethical principles (Beauchamp & Childress, 2013). This comes as no surprise, because bioethics closely resemble digital ethics in the way new forms of agents, patients, and environments are addressed. Thus, bioethical principles meet the ethical challenges caused by AI quite well (Floridi et al., 2018). An ethical approach to AI contributes to solve the tension between leveraging the benefits and preventing or at least mitigating potential harms of AI—a “dual advantage” for society (Floridi et al., 2018, p. 694).

A unifying characteristic of the discourse on AI ethics is the focus on high-level ethical principles and little reference to philosophical ethical theories (Stahl et al., 2021). However, the suitability and practicability of a predominantly principled approach is called into question (e.g., Hagendorff, 2020; Mittelstadt, 2019; Theodorou & Dignum, 2020). First, artificial intelligence cannot be considered in isolation, but within the socio-technical system (i.e., people, organizations, their interactions, and processes organizing these interactions) it is operating and unfolding. Therefore, concrete ethical and socio-legal governance and policies are needed (Cath, 2018; Theodorou & Dignum, 2020). Second, the rather deontological, principled approach to AI ethics based on normative, high-level imperatives and principles (Hagendorff, 2020) lacks translation into practice through mid-level norms and low-level requirements taking into consideration the legal, technical, and social circumstances (Mittelstadt, 2019). Among other things, practical guidance on *how* to develop ethical AI is required in order to close the gap between principles (*what*) and practice (*how*). Hence, applied AI ethics are in demand (Morley et al., 2020).

**Fig. 1:**
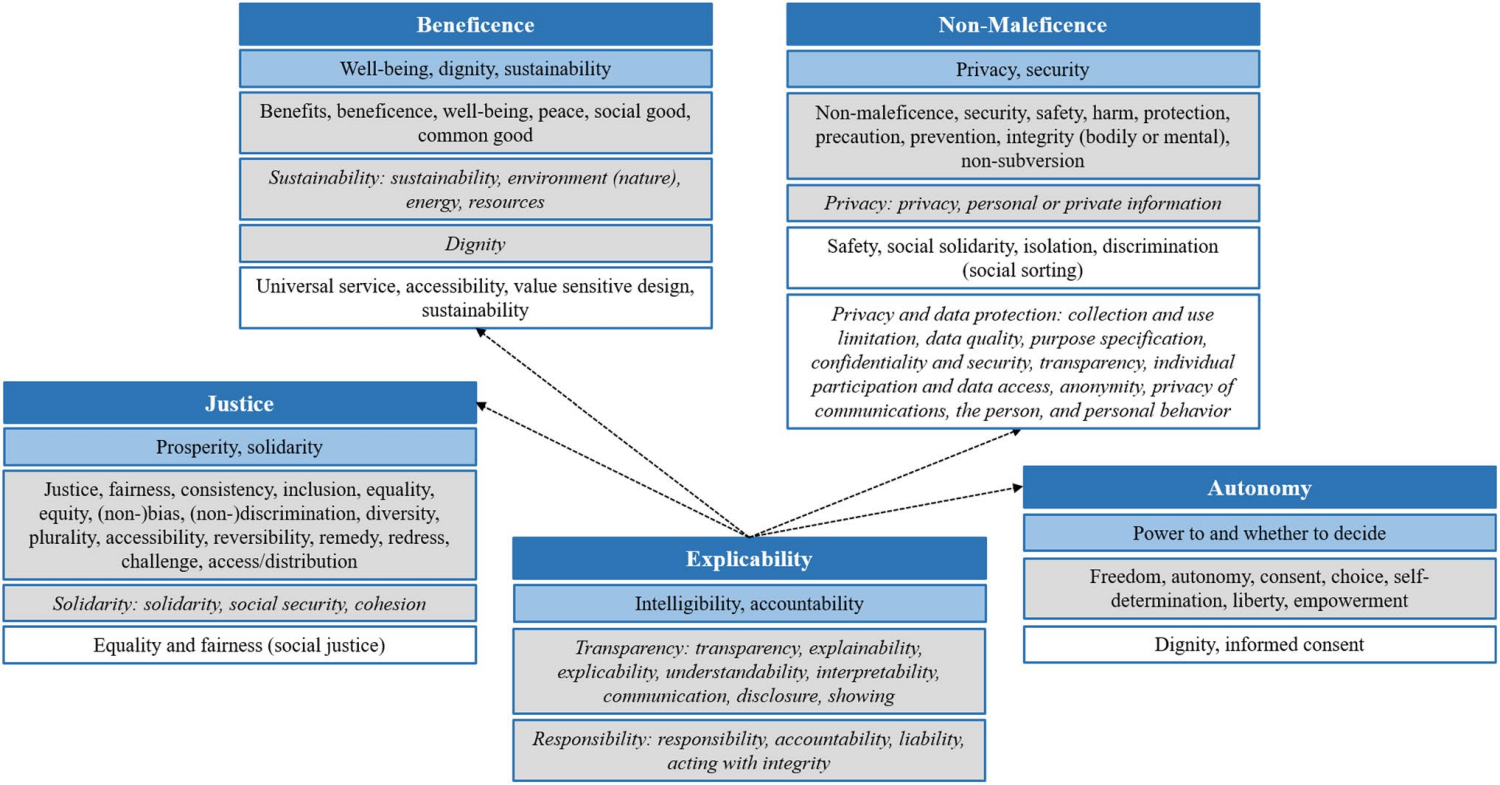
AI ethics map. Principles in blue boxes are taken from Floridi et al. (2018), principles in gray boxes are taken from Jobin et al. (2019), and principles in white boxes are taken from Wright (2011). Principles in *italics* were not subsumed under *beneficence*, *non-maleficence*, *autonomy*, *justice*, or *explicability*, but listed as independent principles by Jobin et al. (2019) and Wright (2011), respectively

Generally, the ethical principles related to AI cover ethical issues in respect to particular features of the technology or the consequences of its use (Stahl et al., 2021), which is in the tradition of computer and (information) technology ethics (e.g., Brey, 2000, 2012; Moor, 1985, 2005; Royakkers et al., 2018; Wright 2011). In the technology ethics context, Moor (2005) proposed a tripartite model for understanding technological revolutions that ranges from the introduction and permeation stages to the power stage. As use intensity, number of users, understanding, and integration into and impact on society increase with these stages, so do the ethical challenges. The ethical issues do not simply result from the number of individuals being affected but from the manifold application opportunities of revolutionary technologies for which ethical policies have not been developed yet (Moor, 2005). Just as the AI ethics literature, the literature on ethical (information) technology incorporates recurring principles and themes including *autonomy*, *justice*, *beneficence* (well-being, common good), *non-maleficence* (avoiding harm and risks), dignity, and privacy (Brey, 2012; Royakkers et al., 2018; Wright 2011). Figure 1 provides a systematization of the principles identified by Floridi et al. (2018); Jobin et al. (2019), and Wright (2011)—the latter to establish the connection to information technology ethics.

Across frameworks, principles and themes are sometimes labeled and subsumed differently. For instance, Floridi et al. (2018) assign solidarity to the *justice* principle, whereas Wright (2011) subsumes solidarity under *non-maleficence*, and Jobin et al. (2019) consider solidarity as a principle on its own. Dignity is another example of deviating principle alignments: an independent principle according to Jobin et al. (2019) versus subsumed under *beneficence* and *autonomy* by Floridi et al. (2018) and Wright (2011), respectively. Moreover, some principles are not entirely distinct or independent and seem to overlap. Particularly, the emphasis of avoiding biases and discrimination under the *justice* principle narrowly relates to avoiding any harm under the *non-maleficence* principle. We will further expand on this relation when discussing the ethics of AI in marketing.

Three other points might need clarification. First, privacy is often referenced as a principle on its own. However, the privacy theme in AI and technology ethics regularly emphasizes that infringements of privacy, breaches of data protection, and misuse of data have to be avoided in the adoption of AI and technology. That is, harms and risks in respect to personal data and privacy have to be limited and prevented, which is pivotal to the *non-maleficence* principle. Therefore, we follow Floridi et al. (2018) and subsume privacy under the *non-maleficence* principle. Second, synthesizing transparency, intelligibility, responsibility, and accountability as *explicability* accounts for the interrelationships of these themes (e.g., Coeckelbergh, 2020; de Laat, 2018; Lepri et al., 2018; Martin, 2019; Morley et al., 2020). Judgments about the responsibility or accountability for AI-based decision outcomes necessitate a certain understanding of the underlying processes leading to these decisions (i.e., transparency or intelligibility). That is, transparency can be a key enabler of and prerequisite for accountability (Lepri et al., 2018). Third, we concur with prior research that considers *trust* as an outcome of ethical principles (e.g., Thiebes et al., 2020) and AI characteristics (e.g., Glikson and Wolley, 2020). Besides, others scholars even claim that “one needs to either change ‘trustworthy AI’ to ‘reliable AI’ or remove it altogether” (Ryan, 2020, p. 2765). Therefore, we refrained from presenting *trust* as an ethical principle in itself. In our following conceptual analysis, we examine the ethical principles and controversies related to AI in the marketing context.

## The Ethics of AI in Marketing

**Fig. 2:**
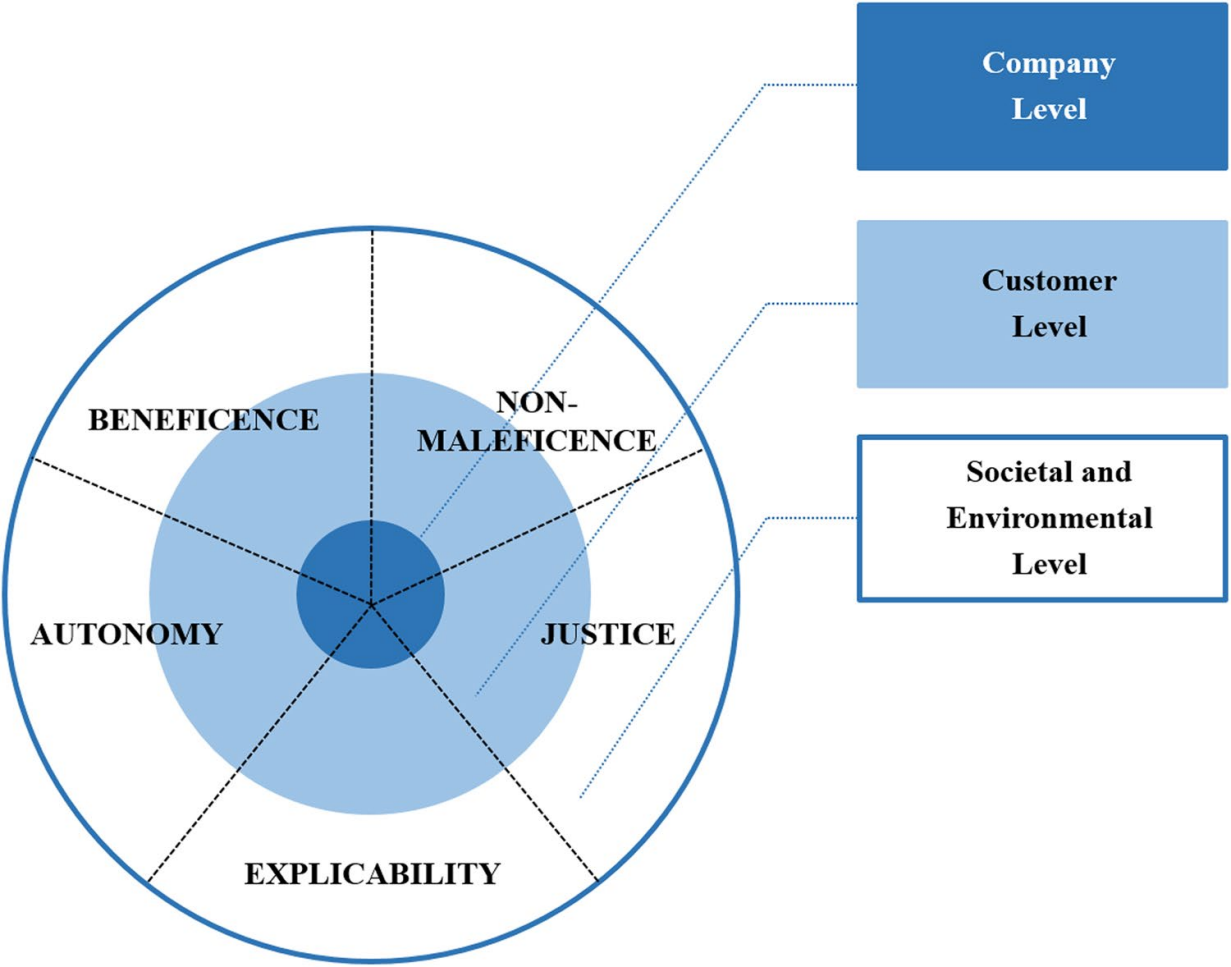
Multi-stakeholder model of AI ethics in marketing

We investigate the ethical implications and concerns of using AI in marketing from the standpoint of multiple stakeholders encompassing the company, customer, and societal and environmental perspectives (see Fig. 2). Thereby, we scrutinize the validity and applicability of ethical principles across different stakeholder levels, and whether tensions between ethical principles emerge due to different stakeholder interests. This multiperspectivity further accounts for the AI-for-social-good perspective stressed by prior AI ethics literature (e.g., Cowls et al., 2021; Floridi et al., 2018, 2020; Taddeo & Floridi, 2018). Correspondingly, we rely our analyses on the applied AI ethics typology suggested by this stream of research, that is, *beneficence*, *non-maleficence*, *autonomy*, *justice*, and *explicability* (Floridi et al., 2018; Morley et al., 2020). Since we aim to provide an epistemological picture on AI ethics in marketing, the list of AI applications we cover does not claim to be exhaustive. We also refrain from providing technical or methodological details on respective AI applications and systems (for brief overviews of different AI methods such as machine or deep learning, see for instance Campbell et al., 2020; De Bruyn et al., 2020; Hagen et al., 2020; Ma & Sun, 2020).

### Beneficence

A focal advantage of leveraging AI in marketing is the opportunity to personalize and customize products and services and the entire marketing mix to maximize engagement, relevance and persuasion, as well as customer satisfaction (Huang & Rust, 2021a, 2021b; Kumar et al., 2019; Puntoni et al., 2021). For example, predicting individuals’ psychological traits from their digital footprints and smartphone data (e.g., Gladstone et al., 2019; Stachl et al., 2020; Youyou et al., 2015) offers substantial opportunities for psychological targeting by crafting psychologically tailored advertising and persuasive appeals (Hagen et al., 2020; Matz & Netzer, 2017; Matz et al., 2017; Matz, Appel, et al., 2019; Matz, Menges, et al., 2019; Matz, Segalin, et al., 2019). Recent research showed that even individuals’ income can be predicted from their Facebook Likes and status updates with an accuracy of up to *r* = 0.43 (Matz, Menges et al., Matz, Appel, et al., 2019). Digital customer data in the form of user-generated content can be further employed to identify customer needs by means of AI (Timoshenko & Hauser, 2019). Furthermore, AI-based recommender systems can benefit both companies and customers. Recommender systems refer to (algorithmic) functions that use information about customer preferences (e.g., products) as inputs to predict how customers would rate certain items under evaluation (e.g., new products available) and how they would rank a set of items individually or as a bundle (Milano et al., 2020). In the case of collaborative filtering as one dominant form of recommender systems, recommendations are based on customers’ past behavior, choices, and preferences, and on preferences of other customers that are structurally similar to them (Cappella, 2017). Recommender systems help e-commerce companies and online retailers to turn browsers into buyers, foster cross-selling, and personalize offerings and browsing experience. On the other hand, customers profit from them through information (pre-)filtering as well as higher quality and efficiency of purchase decisions (e.g., Banker & Khetani, 2019; Lee & Hosaganar, 2019; Lu et al., 2015; Shankar, 2018).

In sum, both companies and customers can gain from personalized recommendations, products, services, persuasive appeals, and marketing mix in general. These supply- and demand-side benefits result from better addressing and serving customers’ cognitive and affective needs and preferences on the one hand and resource efficiency (i.e., time and costs) on the other hand. Thus, *beneficence* on the company and customer levels can be assumed. Besides, the use of AI in marketing is linked to a clear benefit and not developed and employed for the sake of it. In other words, it has a clear purpose, which implies justification as one requirement for *beneficence* (Morley et al., 2020). Of course, one has to point out that the notion of goodness, which is at the core of the *beneficence* principle, is far from being objective both on the individual and superordinate levels (D’Acquisto, 2020). On the individual (customer) level, predictions of future choices based on patterns of customers’ past choices and preferences of similar other customers through recommender systems can be considered as a surrogate for social influence (Cappella, 2017). Customers’ evaluations of recommender systems’ *beneficence* might differ (or worsen) if they would be aware of these indirect social influences by understanding recommender systems’ underlying processes and functionalities (i.e., intelligibility). However, black box AI can be an obstacle to such an understanding.

Potential irreconcilability of what is deemed good for individuals (e.g., customers) and what goodness means on a superordinate level (e.g., society, environment) tends also to arise in the case of AI applications in marketing. That is, the unconditional *beneficence* of these AI applications can be questioned on the societal and environmental level.

In essence and simplified terms, AI applications in marketing pursue sales objectives and increase consumption. While consumption satisfies needs and is thus beneficial on the individual (customer) level (e.g., Csikszentmihalyi, 2000), it simultaneously deplete resources, negatively impacts the environment, and drives climate change (e.g., Swim et al., 2011; Wiedmann et al., 2020). Consumption externalities, which are not self-correcting and inhibit growth, emerge (Dasgupta & Ehrlich, 2013). For instance, clothing consumption, partly due to the rise of fast fashion models, results in a tremendous environmental impact of the fashion industry and its supply chain operations. It ranges from 79 trillion liters of water consumption per year and 4–5 billion tons CO_2_ emissions annually (8–10% of global CO_2_ emissions) to over 92 million tons of textiles waste per year (Niinimäki et al., 2020). These issues could be aggravated by recommender systems and inherent information exploitation versus exploration strategies. For example, Amazon, whose e-commerce platform relies on AI-driven recommender systems and collaborative filtering (Milano et al., 2020), had a relative carbon footprint of 122.8 g of CO_2_ equivalents (including packaging, transportation, purchased electricity, fossil fuel emissions from direct operations etc.) per dollar of gross merchandise sales in 2019 (Amazon, 2020) and net sales of $386.1 billion in 2020 (Amazon, 2021). Even if one assumes that both the relative carbon footprint (due to decarbonization efforts and investments) and sales (due to customers’ return to offline channels and shopping when the Covid-19 pandemic will impose less restrictions) might decrease in 2021, the carbon footprint of the world’s largest e-commerce company amounts to dozens of tons of CO_2_ emissions annually. Generally, e-commerce and online retailing can have more detrimental environmental impacts and larger ecological footprints due to packaging, product returns, last mile transportation, and shopping basket sizes as compared to traditional retailing and shopping (e.g., Escursell et al., 2021; Pålsson et al., 2015; Shahmohammadi et al., 2020; van Loon et al., 2015). Moreover, AI-enabled personalized mobile marketing (e.g., Tong et al., 2020) and in-store communication and technology (e.g., Dekimpe et al., 2020; Grewal, Noble, et al., 2020; van Esch et al., 2021) can prompt unplanned offline purchases and impulsive buying, which, in turn, amplify consumption and its environmental drawbacks. Finally, information and communication technologies, applications, and systems related to AI themselves can have rebound effects caused by energy consumption and emissions of AI development, production, and deployment (e.g., Belkhir & Elmeligi, 2018; Dhar, 2020; Lange et al., 2020).

On the individual level, exploitation of customer information that AI systems already possess constitutes the optimal (standard) strategy to maximize individual utility by satisfying preferences. Conversely, AI systems’ exploratory recommendations of new alternatives (e.g., sustainable items) might be the strategy with greatest expected utilities on a societal level (Milano et al., 2021). The environmental impact and material footprint of consumption (Wiedmann et al., 2015) that could be additionally fueled by AI applications in marketing contravene the *beneficence* principle of promoting well-being of humans and the planet. The negative externalities further establish the connection to the *non-maleficence* principle that advocate the prevention of any risk and harm due to overuse or misuse of AI (Floridi et al., 2018).

### Non-Maleficence

In terms of the potentially adverse consequences of intensified, AI-driven consumption on the societal and environmental level, the judgments of *beneficence* and *non-maleficence* are akin. That is, AI applications do not necessarily advance the environmental good (*beneficence* principle not met) but can impair it (*non-maleficence* principle not met). In contrast, these ethical judgments do not coincide on the company and customer levels. That means, AI applications could be beneficent and maleficent at the same time (Milano et al., 2021).

Of particular importance in respect to *non-maleficence* of AI are personal privacy, accuracy, as well as data protection and quality (e.g., Floridi et al., 2018; Morley et al., 2020). Privacy risks can arise (1) when data are collected without informed consent of customers, (2) after storage when they are leaked or de-anonymized (i.e., data breaches), or (3) when AI systems draw inferences from both the individual customer data (directly) or interaction data with other customers (indirectly). The latter particularly pertains to collaborative filtering (Milano et al., 2020). Given the extensive use of customer information and data-driven, analytical approaches in marketing, marketing scholars and practitioners have been and are highly concerned with privacy issues (e.g., Bleier et al., 2020; Martin & Murphy, 2017; Martin & Palmatier, 2020; Martin et al., 2020; Okazaki et al., 2020; Stewart, 2017; Thomaz et al., 2020). The massive amounts of data feeding AI systems and applications potentiates privacy and data protection issues, as discussed for algorithms (e.g., Mittelstadt et al., 2016), recommender systems (e.g., Milano et al., 2020), and psychological targeting (e.g., Matz, Appel, et al., 2019; Matz, Menges, et al., 2019; Matz, Segalin, et al., 2019), among other things. Supranational regulations such as the European Union’s General Data Protection Regulation (GDPR) attempt to counter these issues by requiring data protection impact assessment (Art. 35 GDPR) and data protection by design and by default (Art. 25 GDPR). The latter stipulates that companies have to proactively integrate privacy protection into the design, development, and application of data-driven technologies and to set privacy defaults to reasonable levels of protection (Andrew & Baker, 2021; Matz, Appel, et al., 2019; Matz, Menges, et al., 2019; Matz, Segalin, et al., 2019).

Since ethical issues related to privacy and data protection are extensively discussed elsewhere (e.g., Floridi & Taddeo, 2016; Mittelstadt & Floridi, 2016), we want to briefly hint at the tension and potential tradeoff between the scale and scope of data capture and privacy concerns. That means that predictive validity and accuracy of AI predictions increase with the amount of input data, which, however, could interfere with data protection and privacy. In the marketing context, personalization of marketing measures and customer privacy trade off (Rust, 2020). Accuracy of results predicted by AI applications also rests upon the quality and integrity of data. AI systems’ inferences are as reliable as the underlying data. Biases, inaccuracies, errors, and mistakes inherent in the data could lead to biased results and false conclusions (Barredo Arrieta et al., 2020; Morley et al., 2020), which, in turn, could be maleficent for both customers and companies (e.g., Banker & Khetani, 2019). Besides, algorithmic decisions based on (potentially spurious) correlations found in large data sets could be problematic. That can be the case when causality is not established prior to actions, and actions are then directed to individuals, although the knowledge generated concerns populations (Mittelstadt et al., 2016). Inferior predictions and recommendations can be particularly adverse for customers if they depend too much on algorithm-generated recommendations that could then diminish their well-being (Banker & Khetani, 2019). Banker and Khetani (2019) refer to this phenomenon as algorithm overreliance and frame it as a type I problem (false positive). That means that false propositions (i.e., inferior recommendations) are incorrectly deemed true (i.e., customers adopt recommendations). Following this classification, type II problems (false negative) could negatively impact companies (e.g., cross-selling), since customers favor their own intuitions and do not adopt superior algorithm-based recommendations. This (behavioral) phenomenon has been conceptualized as algorithm aversion (e.g., Dietvorst et al., 2015, 2018). Reliance on algorithms can not only impact companies indirectly through their customers, but also directly when used for setting real-time prices for products and services (Hansen et al., 2021). Tacit collusions among algorithms—that is, neither communication between algorithms nor ex ante design or instruction to collude—(e.g., Calvano et al., 2020) or misspecified models overestimating price sensitivities (e.g., Hansen et al., 2021) can make algorithms charge supra-competitive prices. That eventually harms companies in their entirety, customers, and at worst society at large due to increasing overall price levels.

Again, black box AI in respect to the extent of data capture, data quality, model specification, among other things, complicate the judgments of *non-maleficence* and the decision of whether to rely on algorithmic predictions for both customers and companies. While the perspective on algorithm-driven decision-making focuses on decision outcomes, the *autonomy* principles rather entails the process view of decision-making.

### Autonomy

Consumer autonomy is central to consumer choice and defined as “consumers’ ability to make and enact decisions on their own, free from external influences imposed by other agents” (Wertenbroch et al., 2020, p. 430). In the AI ethics context, *autonomy* relates to a meta-autonomy or decide-to-delegate model. That is, “humans should always retain the power to decide which decisions to take” on their own or when to cede decision-making control (Floridi et al., 2018, p. 698). In general, freedom of choice and self-determination can be seen as intrinsic good or right contributing to individuals’ well-being (Burr et al., 2020). Human agency (i.e., autonomous decisions) and human oversight are focal requirements of *autonomy* in relation to AI applications (Morley et al., 2020).

On the company level, governance mechanism should be implemented to keep humans in the loop (e.g., Thiebes et al., 2020). That is particularly important when AI systems are operating in ethically or morally salient contexts (e.g., Jotterand & Bosco, 2020), which could be the case for developing and deploying feeling AI (Huang & Rust, 2021b) or humanized AI (Kaplan & Haenlein, 2019) in marketing. To date, the development of artificial moral agents is still in its infancy (e.g., Cervantes et al., 2020). However, it is not unlikely that future (emotionally intelligent) AI systems and applications in service and customer relationship management will encounter moral decisions, ethical issues, or emotionally charged customer interactions. To effectively apply AI in the latter situation necessitates AI systems’ understanding of emotions beyond simple recognition. Until AI systems turn from “psychopaths” recognizing and faking emotions into emotionally intelligent and moral agents, human agency will be crucial (De Bruyn et al., 2020, p. 96).

On the customer level, AI applications can shape decision-making processes. Personalization, psychological targeting, and recommender systems can serve as adaptive, structural, or informational nudges (Burr et al., 2018; Floridi, 2016; Milano et al., 2020; Sunstein, 2016). These kind of interventions influence customers’ choice sets—the choice architecture (Thaler & Sunstein, 2008) -, or information related to choices and eventually preferences and decisions. Customers’ autonomy is impacted in such a way that decisions are delegated to AI systems at the information collection stage of the decision-making process, particularly, (pre-)filtering of information and options customers are exposed to. That can be beneficial due to resource efficiency (e.g., time, cognitive resources) and tailored content (Burr et al., 2018), but also detrimental to customers in case of aversion of or overreliance on AI systems’ recommendations (e.g., Banker & Khetani, 2019; Dietvorst et al., 2015, 2018) or due to manipulated or deceptive content (e.g., Burr et al., 2018; Milano et al., 2020). An informed and conscious decision of humans (as customers or company representatives) of whether to delegate their decision-making power to AI systems and applications would again demand a certain degree of understanding of AI functionalities (i.e., intelligibility).

### Justice

Human judgments can be biased and discriminating, and so the predictions of AI applications and algorithms constructed by humans can be biased and result in discrimination as well (Kleinberg et al., 2018, 2020). AI can fall victim to the same errors and biases that humans do, reproduce, and amplify them (Rich & Gureckis, 2019). Among other things, AI-powered personalization strategies in marketing (e.g., Huang & Rust, 2021b), psychological targeting (e.g., Matz & Netzer, 2017; Matz et al., 2017; Matz, Appel, et al., 2019; Matz, Menges, et al., 2019; Matz, Segalin, et al., 2019), and customer prioritization in customer relationship management (e.g., Libai et al., 2020) could discriminate certain customer groups against others on the basis of demographic, psychological, and economic factors. Particularly, gender, age, and racial disparities, prejudices, and stereotypes can be reinforced by AI systems and applications (Bol et al., 2020; Datta et al., 2015; Lambrecht & Tucker, 2019; Obermeyer et al., 2019). Moreover, targeting of vulnerable customer groups or prioritization in respect to income or profitability can be problematic, aggravate existing inequalities, or harm customers (e.g., Libai et al., 2020; Matz & Netzer, 2017; Matz et al., 2017). Personalization of offerings or the whole marketing mix could “segment a population so that only some segments are worthy of receiving some opportunities or information, re-enforcing existing social (dis)advantages” (Mittelstadt et al., 2016, p. 9). As mentioned above, biased AI predictions, unfair and unequal treatments and targeting can result from biases in and skewness of underlying data (Barredo Arrieta et al., 2020; Morley et al., 2020). Biased and skewed data can be due to over- and underrepresentation of certain demographic groups or sensitive features, inclusion of misleading proxy features (Barredo Arrieta et al., 2020), or sparse (small) data for certain individuals/groups, phenomena, and features (Rich & Gureckis, 2019). Imbalances in customer data and corresponding data- and AI-driven discrimination and biases can also stem from customers’ increasing unwillingness to share data online and/or with companies due to privacy concerns (Du et al., 2021). Finally, endogeneity in data and feedback loops can bias AI predictions. That is, (biased) data lead to (biased) predictions that inform decisions, which, in turn, serve as data inputs (De Bruyn et al., 2020). In light of these multiple sources of biases, diligence and monitoring along the entire data lifecycle and in respect to AI development (e.g., model specification) are advisable if not indispensable. Therefore, marketers, data scientists, and AI developers could team up with ethicists. The “gold standard … would be an ethicist … as a dedicated member of the development team” (McLennan et al., 2020, p. 488).

Taken together, customers can be differently targeted and affected by AI. Consequent discrimination and amplification of existing inequalities can, in turn, diminish social good and well-being, which establishes the connection to the *beneficence* and *non-maleficence* principles. For the sake of completeness, we also have to note that recent research showed that algorithms can be employed as discrimination detectors and to de-bias human judgments (Kleinberg et al., 2018, 2020).

On the company level, recommender systems can discriminate firms by decreasing the variety of products consumers explore and purchase (i.e., sales diversity) and by increasing market share concentration for popular products (Lee & Hosaganar, 2019). Besides, the rise of multisided e-commerce platforms deploying AI-generated recommendations can endanger traditional retailers that do not participate in these platforms by undermining their business models and impeding market access (Milano et al., 2021).

As in the case with the *non-maleficence* principle, black box AI can obfuscate biased inputs and outputs of AI systems and thus hamper judgments of *justice*.

### Explicability

*Explicability* might be the most prevalent and controversial principle of AI ethics due to the black box nature of AI systems, their opacity, and lack of accountability (Ananny & Crawford, 2018; Milano et al., 2020; Mittelstadt et al., 2016; Rai, 2020; Rudin, 2019; Thiebes et al., 2020). Correspondingly, calls for interpretable and explainable AI (XAI) are growing louder, particularly, when high-stake decisions and sensitive, personal data are involved (Barredo Arrieta et al., 2020; De Bruyn et al., 2020; Gunning et al., 2019; Proserpio et al., 2020; Rai, 2020; Rudin, 2019). In the literature, different nomenclature and concepts including intelligibility, comprehensibility, interpretability, explainability, and transparency are used interchangeably and inconsistently (Barredo Arrieta et al., 2020), and are partly misconceived (Rudin, 2019). For instance, Rai (2020) defined XAI as “the class of systems that provide visibility into how an AI system makes decisions and predictions and executes its actions” (pp. 137–138). On the other hand, Rudin (2019) conceptualized XAI as a second (post hoc) model to explain the initial first black box model and thus advocated inherently interpretable models (i.e., interpretable AI) instead of XAI. De Bruyn et al. (2020) summed up explainable and interpretable AI as methods to explain AI systems’ intentions, data inputs and sources, and the relation between inputs and outputs, so that results such as predictions, classifications, and recommendations could be understood by human experts. In a comprehensive review, Barredo Arrieta et al. (2020) identified intelligibility (i.e., human understanding of a model’s function without any need for explaining its internal structure or underlying data processing algorithm) as the focal concept related to XAI. This finding further corroborates the focus on intelligibility by Floridi et al. (2018). Given that intelligibility can have an enabling function for the other ethical principles, a lack thereof (i.e., black box AI) could impede individuals’ judgments about *beneficence*, *non-maleficence*, *justice,* and *autonomy*. That could particularly shape individuals’ self-determination and decisions about whether to delegate decisions to AI systems at all (i.e., meta-autonomy).

For customers, simple explanations of how AI works (i.e., intelligibility as the epistemological dimension) might be more effective and satisfying than complicated ones causing information overload, irritation, and frustration (Rai, 2020). In light of direct or indirect consequences of AI-based personalization, targeting, and recommendation applications, customers have a legitimate interest in knowing who to hold accountable (i.e., accountability as the ethical dimension) for adverse, biased, or discriminatory outcomes of AI predictions. This will acquire increasing importance if AI systems and algorithms are considered and conceptualized as value-laden rather than neutral (e.g., Martin, 2019). That, again, relates to potential biases of AI developers, the corresponding need of a certain degree of human oversight, and hence to the *autonomy* and *justice* principles. Eventually, companies should be responsible for the AI systems they develop and deploy and obliged to deal with the respective ethical implications or challenges.

Concepts remedying the black box issue of AI are not undisputed. For instance, transparency can interfere with privacy concerns and proprietary boundaries aiming at facilitating exclusivity or competitive advantages. Furthermore, transparency can be subject to cognitive (i.e., information overload, lack of understanding), technical (i.e., methodological and technical complexity), and temporal restrictions (i.e., rapid advancements and development cycles) (Ananny & Crawford, 2018). Moreover, explainable AI methods could provide explanations that are too complicated for humans to comprehend, not faithful to what original models compute, or insufficiently detailed to understand what models are doing (Rudin, 2019). Besides, transparency and disclosure of AI identities can compromise performance and efficiency of AI systems such as bots (e.g., customer purchases). That raises the question whether intelligent machines should hide their non-human nature for the sake of efficiency and how to weigh costs and benefits of transparency (Ishowo-Oloko et al., 2019; Luo et al., 2019). Finally, explaining customer why they received certain recommendations—for example, since the item presented is the most popular one among users—might amplify desirability of a choice alternative and reinforces its popularity. Thereby, variety of alternatives and plurality of choices can decline, which hampers competition and negatively affects companies (Milano et al., 2020). Our preceding assessment reveals several interdependencies between ethical principles, which we illustrate subsequently.

### Interdependencies Between Ethical Principles and Levels of Intelligence

**Fig. 3:**
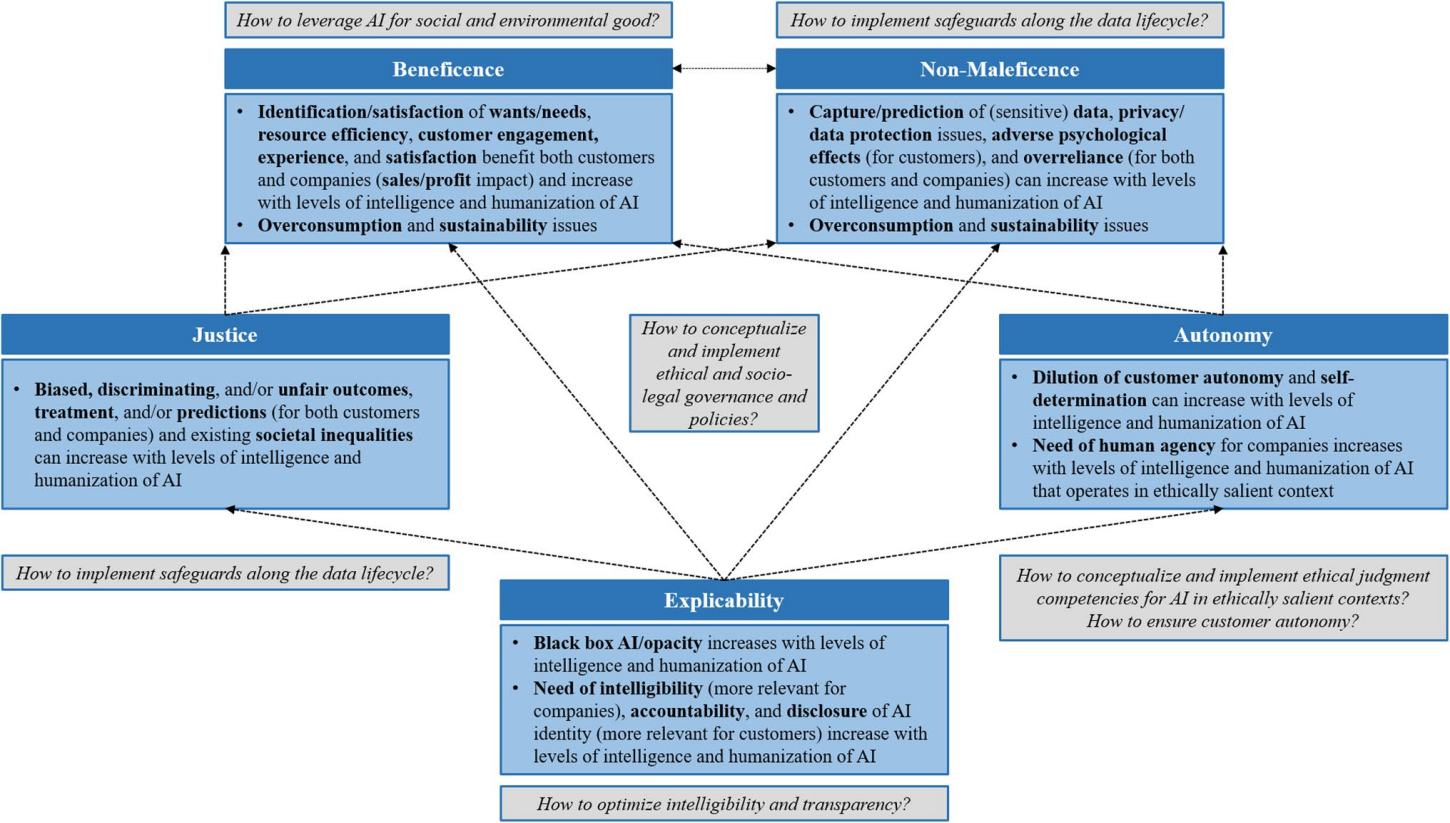
Interdependencies of ethical principles and levels of intelligence and future research questions

As our conceptual analyses have shown, ethical principles related to AI in marketing interact and collide and thus cannot be judged in isolation, but in relation to each other. First, *explicability* can be considered as enabling principle for *beneficence*, *non-maleficence*, *justice*, and *autonomy*, while the latter two determine *beneficence* and *non-maleficence* as well (see Fig. 3). Second, AI systems and applications can be beneficent and maleficent at the same time, depending on which stakeholders are concerned. Even an inverse relation between *beneficence* and *non-maleficence* does not seem unlikely. For example, increased AI-based personalization strategies satisfy customer needs (customer benefit) which increases company sales (company benefit), but also raises the aggregate consumption level, which harms the environment and society at large (societal and environmental harm). Furthermore, the benefits of personalization for both customers and companies (whose potential might increase with the amount of customer data) can be compromised by privacy and data protection issues (e.g., Rust, 2020). Under certain conditions, AI applications can be both beneficent and non-maleficent. Assuming that sensitive health data are gathered after obtaining informed consent, treated confidentially, and used conscientiously, medical AI (e.g., Longoni et al., 2019; Yun et al., 2021) has the potential to promote individual well-being and preventing harm, which, in turn, benefits health care systems and thus society at large. Besides, customer service robots can ameliorate customer experience and reverse information technology service work offshoring (i.e., through botsourcing) while addressing the technology inequality phenomenon by reducing access barriers for seniors (Xiao & Kumar, 2021).

Ethical issues related to AI are not static and uniform but differ across areas of application, technological sophistication, and pervasiveness. Following Moor (2005), we assume that ethical challenges and tensions (can) increase with the level of intelligence and degree of humanization of AI (see Fig. 3). As conceptualizations of the AI development stages are multi-faceted and deviate (Davenport et al., 2020; Huang & Rust, 2021b; Kaplan & Haenlein, 2019), they are also subject to temporal change (Stahl et al., 2021), and transitions between stages are rather fluid. Therefore, we simplistically refer to increasing levels of intelligence capturing the transition from cognitive (analytical, mechanical) to emotional (feeling) and social intelligence of AI systems and applications and an increasing degree of humanization. We suggest that *beneficence* of AI increases with the level of intelligence and humanization of AI (e.g., targeted customer need identification and satisfaction), but so do the issues related to *explicability* (e.g., black box AI, accountability in case of failures). Ethical challenges in respect to *justice* and *autonomy* can increase, but do not necessarily have to. For instance, AI could serve as discrimination detectors (Kleinberg et al., 2018, 2020). However, the need for human agency and oversight is assumed to increase, particularly, when (rather opaque) AI is operating in ethically salient contexts. Whether *non-maleficence* will be achieved in the future depends on the extent of customer data gathering and treatment of sensitive data, among other things. Increasing levels of emotional/social intelligence and humanization of AI are related to anthropomorphism (Epley et al., 2007; Kim et al., 2019; Waytz, Cacioppo, et al., 2010; Waytz, Gray, et al., 2010) as well as mind perception of and attribution of experience/warmth and agency/competence to AI (e.g., Choi et al., 2020; Fiske et al., 2007; Gray et al., 2007; Waytz, Cacioppo, et al., 2010; Waytz, Gray, et al., 2010). Intense use of and interaction with human-like AI could lead to psychological ownership and emotional attachment (e.g., Morewedge, 2021; Morewedge et al., 2021; Shu & Peck, 2011). Both psychological phenomena might have detrimental psychological effects if AI deployment gets out of control and/or overreliance occurs. In a similar vein, extensive use could end up in (perceived) dehumanization of users (e.g., Castelo et al., 2019; Haslam, 2006; Haslam & Loughnan, 2014).

From a purely deontological perspective, AI applications could be entirely called into question when *non-maleficence* cannot be taken for granted. In consequence, promising opportunities to serve customers and *beneficence* on the customer and company levels would be missed. Thus, a principled, deontological AI ethics approach with normative imperatives and maxims could to be inappropriate to account for colliding principles. A “deontologically inspired tick-box exercise” (Hagendorff, 2020, p. 112) might be ill-suited to scrutinize such far reaching ethical concerns. Instead, a utilitarian perspective weighing benefits and costs across all stakeholders—for instance, utilitarian calculations based on ethical foresight (e.g., Floridi & Strait, 2020)—could complement high-level ethical principles. While the deontological perspective proves valuable in providing guidelines oriented on and aligned to human values, the utilitarian approach could better account for multiple values, objectives, and utilities at the individual, group, and societal levels. AI applications and systems could incorporate multi-objective maximum-expected-utility concepts that are aligned to human values and ethical principles (e.g., Vamplew et al., 2018). The technological design and implementation of such concepts is anything but trivial and challenging on two counts. First, utilities to be optimized are difficult to grasp and determine or differ across stakeholders (Butkus, 2020). Second, the nascent stage of artificial moral agency still demands human agency. Eventually, humans are in charge of determining to which ethical principles and human values AI applications and systems (and their utility functions) should be aligned. Besides, decisions about AI design approaches to equip AI applications and systems with ethical judgments competences have to be taken by humans. Initially, humans have to determine whether AI systems base their ethical decision-making on pre-defined ethical theories (top-down), on more flexible self-learning mechanisms based on certain values (bottom-up), or on a combination of both (hybrid) (Bonnemains et al., 2018; Cervantes et al. 2020). In the following section, we provide thoughts and ideas of how to align AI applications in marketing with ethical principles to promote social and environmental good.

## AI in Marketing for Social and Environmental Good

As delineated above, AI in marketing can additionally fuel consumption due to personalized marketing mix measures, psychological targeting, and effective customer relationship management. However, augmented consumption or overconsumption undermines the transition to sustainability, resulting in calls for sufficiency-oriented lifestyles and to “consume better but less” (Wiedmann et al., 2020, p. 4). Likewise, approaches focusing sufficient consumption (e.g., Gossen et al., 2019), mindful consumption (e.g., Bahl et al., 2016; Sheth et al., 2011), sustainable consumption (e.g., White et al., 2019) and marketing (e.g., Hunt, 2011; Sheth & Parvatiyar, 2021), and consumption ethics (e.g., Carrington et al., 2021) have become the focus of scholarly attention. In this context, White et al. (2019, p. 23) argued that “marketing and sustainability are inextricably intertwined”, although goals and assumptions of both seem incompatible at first sight. AI can be a powerful force in reconciling marketing activities with sustainability and social good objectives.

Data-driven segmentation and targeting approaches can bring together tailored persuasive appeals and offerings and consumers in relation to their predisposition to ethical and sustainable consumption and products. The sophistication and computational power of AI applications allow to account for the complex interrelationships between supply-side (e.g., marketing mix) and demand-side factors (e.g., consumer demographics and personality, decision-making processes) in respect to ethical and sustainable consumption. For instance, price, product, and sustainability attributes are differentially valued in dependence of consumers’ self-other orientations (Ross & Milne, 2020). Moreover, the purchases of sustainable products using green-identity labels can decrease when combined with price discounts (Schwartz et al., 2020). Brands’ ethical attributes and strengths (i.e., cause-related marketing and corporate social responsibility) further exert stronger influences in the choice phase than in the consideration (set formation) phase of the consumer decision journey (Schamp et al., 2019). In general, a multitude of social, structural, and individual factors can strengthen or attenuate consumers’ sustainable consumption intentions and behavior (White et al., 2019). Particularly, psychological factors can become barriers to behavioral change and “dragons of inaction” (Gifford, 2011, p. 290).

This anecdotal evidence already alludes to one opportunity to harness AI for societal and environmental well-being, that is, psychological targeting. Psychological targeting facilitates to identify consumers predisposed to sustainable and ethical products and attributes based on their psychological traits and to tailor respective persuasive appeals (e.g., Matz, Appel, et al., 2019; Matz, Menges, et al., 2019; Matz, Segalin, et al., 2019), for example, by emphasizing specific product attributes or using green-identity and eco labels. Given the possibility to predict income from digital footprints (e.g., Matz, Menges et al., Matz, Appel, et al., 2019), marketers can also personalize prices by deriving consumers’ willingness to pay for environmentally friendly products. That also helps to streamline promotion and price policies, particularly, against the backdrop of the moderating effect of price on the relation between green-identity labeling and consumer behavioral responses (Schwartz et al., 2020). These opportunities relate to the stance of promoting social and environmental good (i.e., *beneficence*), but marketers are also obliged to avoid misuse of psychological targeting and corresponding harm to individuals (i.e., *non-maleficence*). Therefore, marketers should avoid targeting vulnerable groups or consumers who are prone to addictive and compulsive behaviors (Matz & Netzer, 2017) or compulsive buying, which can be inferred from psychological factors (e.g., O’Guinn & Faber, 1989). As aforementioned, discriminatory treatment of individuals and groups by AI systems such as targeting vulnerable groups can arise from biased or skewed underlying data and/or misspecified models (Barredo Arrieta et al., 2020; Morley et al., 2020). To prevent such unfavorable (i.e., *non-maleficence*) and unfair (i.e., *justice*) outcomes that undermine the beneficent purpose of AI applications, marketers should gain a certain understanding of data inputs (e.g., features taken into account or data distributions) and AI functionalities (i.e., intelligibility). Marketers’ awareness of potentially biased data or AI models is a crucial step of this non-trivial task, since they are likely to be held accountable (Huang & Rust, 2021b). Data scientists and AI developers should contribute by leveraging their methodological expertise to identify and correct biases and errors.

As personalized, psychologically tailored messages and appeals can serve as nudges, so can recommendations generated by AI systems for consumer decision-making processes (e.g., Burr et al., 2018; Milano et al., 2020). Basically, AI-based recommender systems should follow the same rationale as psychological targeting. Based on comprehensive purchase (history) and user data, recommendations could nudge consumers to sustainable products or at least offer sustainable alternatives to conventional products. That is, recommender systems should balance exploration of (new sustainable) items and exploitation of existing preferences for their recommendations (Milano et al., 2021). In this way, diversity of product offerings could be increased in terms of environmental friendliness, material footprint, and sustainability (e.g., fast- vs. slow fashion items). Furthermore, customer data can be leveraged to mitigate compulsive buying tendencies (i.e., daily or highly frequent purchases, repeated purchases of comparable or identical products etc.) by providing respective notices to customers. For this purpose, interventions in the form of *nudges to reason* could be applied. These informational nudges do not affect behavior directly, but attempt to change minds by increasing individuals’ responsiveness to genuine evidence (Levy, 2017). Such evidence can take the form of underlining the individual or environmental consequences of excessive or compulsive buying. Stressing the impact of certain purchase decisions can spur consumer to rethink consumption patterns and account for social norms, social desirability, and system justification beliefs (e.g., Gifford, 2011; White et al., 2019). Comparably, recent prior research revealed that making consumers reflect their personal possessions and recall their recent use of it can diminish the desire to shop impulsively (Dholakia et al., 2018).

To minimize interferences with customers’ *autonomy*, informational nudges (i.e., changes of the nature of information consumer are exposed to) should be preferred over structural nudges (i.e., changes of the choice architecture consumers are exposed to), although the former are less successful than the latter (Floridi, 2016). In general, the AI applications illustrated above suggest that the development, design, and deployment of AI should focus on the creation of new opportunities and capabilities to foster societal and environmental well-being. That is the core of the positive computing perspective (Burr et al., 2020). Whether ethics have to be embedded in the design process in a rather structural (i.e., ethics by design) or informational way (i.e., pro-ethical design) (Floridi, 2016) does not only depend on *autonomy* and accountability (i.e., *explicability*) if AI is misused or fails. It is also dependent on the subjective nature of well-being (and utilities) and eventually on customers’ acceptance and behavioral responses. An embedded ethics approach guiding AI developers of how to translate ethical principles into practice through ethics training and exchange with ethicists (Brey, 2000; McLennan et al., 2020; Moor, 2005) should be contemplated to take on this challenge.

## Directions for Future Research

The AI-for-social-good perspective in marketing opens up various research opportunities (see Fig. 3). First, future research should examine which degree of intelligibility and transparency of respective AI applications and systems best matches the tension between customers’ cognitive abilities (e.g., Rai, 2020), information needs, and potential tradeoffs between disclosure of non-human nature and efficiency/performance of AI (e.g., Ishowo-Oloko et al., 2019; Luo et al., 2019; Rai, 2020). Second, safeguards along the entire data lifecycle as well as for AI development and deployment to minimize the risk of biased data and AI predictions and treatments should be investigated. Embedded ethics approaches (e.g., McLennan et al., 2020) that bring together marketers, data scientists, AI developers, and ethicists seem promising. However, appropriate and efficient organizational governance structures and decision-making processes might be in demand. Partly or fully automated internal auditing mechanisms (e.g., Floridi et al., 2018; Rahwan, 2018) could serve as monitoring measures and both internal and external compliance evidence. Third, the *autonomy* principle raises research question on at least two counts. Assuming that the *whether* is a matter of time, companies and AI developers have to craft policies of *how* to endow AI with (a certain) degree of ethical judgment competence for ethically salient contexts, decisions, and customer interactions (e.g., health care robots, emotionally intelligent AI in general). Besides, customers have to retain their decisional power and *autonomy*. In other words, AI-created choice architectures should be free from coercive, deceptive, and paternalistic structures. That might be even more important when it comes to consumption and purchases that are personally relevant or for which humans are deemed more competent than artificial agents (e.g., Granulo et al., 2021; Longoni & Cian, 2021). If AI-shaped customer decisions and behavior in the aggregate take effect on societal and environmental level, humans and even society have to be kept in the loop (e.g., Rahwan, 2018). Finally, on a national or supranational level, binding ethical and socio-legal governance and policies (e.g., Cath, 2018; Stahl et al., 2021; Theodorou & Dignum, 2020) have to be conceived to commit companies to develop and deploy AI in an ethical way in order to promote social good while preventing any harm. Otherwise, one could run the risk of “creating a supermarket of principles and values, where private and public actors may shop for the kind of ethics that is best retrofitted to justify their behaviours” (Floridi, 2019, p. 262).

## Conclusion

This paper synthesizes the mounting research areas of AI ethics and AI in marketing. By scrutinizing the validity of ethical principles related to AI in the marketing context and across stakeholders, we show that ethical principles interdepend and collide, partly, in dependence of the stakeholders concerned. Particularly, *beneficence* and *non-maleficence* cannot be taken for granted, since the advances of AI applications in marketing are likely to increase individual and aggregate consumption. Besides, *explicabilit*y (i.e., intelligibility and accountability) turns out to enable the other ethical principles. Ethical challenges and interdependencies between ethical principles might increase with levels of intelligence and humanization of AI. Thus, a principled, deontological approach to AI ethics, which implies to refrain from AI applications contravening ethical principles, does not account for the complexity of the future AI development and pervasiveness from an ethical perspective. Therefore, we suggest to complement ethical considerations of AI in marketing by a utilitarian perspective balancing benefits and costs.

In essence, ethical principles should not pursue the objective of inhibiting actions or (technological) progress; they should rather amplify the scope of action, autonomy, freedom, and self-responsibility (Hagendorff, 2020). We follow this path and provide ideas of how to leverage AI applications in marketing to promote social and environmental good. Kaplan and Haenlein (2020, p. 44) noted that “AI can be major game changer” to address climate change. We concur with this thought and attempted to show how to add the fuel of AI to the fire of sustainability efforts in the marketing context. To achieve a dual advantage for society (Floridi et al., 2018), this *beneficence*-inspired view is complemented by cautioning against misuse of AI, particularly, when directed at vulnerable consumers. We hope that some of our suggestions motivate marketing researchers and practitioners to further investigate how the AI-powered promotion of well-being can be refined, advanced, and effectively put into practice.
